# Working Together With Consumers to Develop Falls Prevention Education for Older People Receiving Homecare – a Community‐Based Participatory Study

**DOI:** 10.1111/hex.70948

**Published:** 2026-10-26

**Authors:** Jacqueline Francis‐Coad, Jo‐Aine Hang, Vanessa Jessup, Sharmila Vaz, Elissa Burton, Cheng Yen Loo, Bethwyn Joy, Anne‐Marie Hill

**Affiliations:** ^1^ School of Health and Clinical Sciences The University of Western Australia Crawley Western Australia Australia; ^2^ Western Australian Centre for Health & Ageing University of Western Australia Perth Western Australia Australia; ^3^ School of Health Sciences The University of Notre Dame Australia Fremantle Western Australia Australia; ^4^ School of Allied Health and enAble Institute Curtin University Bentley Western Australia Australia; ^5^ Brightwater Research Centre Brightwater Care Group Inglewood Western Australia Australia; ^6^ (Consumer Author) Juniper, Juniper Central Balcatta Western Australia Australia

**Keywords:** accidental falls, aged, co‐design, community participation, community‐based participatory research, consumers, education

## Abstract

**Introduction:**

Frail, older people receiving homecare services have a 50% higher rate of falls compared with age‐matched peers. Falls prevention education can assist older people in understanding risks and preventing falls, but it needs to be relevant and appropriate for both consumers and context. Consumer experience and perspectives can shape decisions that improve programme relevance and health outcomes. This study aimed to revise a novel falls prevention education programme with older homecare clients (consumers) and staff (stakeholders) for implementation in the homecare setting.

**Methods:**

Mixed methods design using a community‐based participatory approach. Consumer and stakeholder focus groups were undertaken using a co‐design framework. Qualitative data were analysed using deductive content analysis and the revised resources were scored using the quantitative Patient Education Materials Assessment Tool.

**Results:**

Twelve consumers and eleven stakeholders (*n* = 23) participated in revising the education programme and valued the opportunity to engage in co‐design. Consumers were aware of environmental risk factors and strategies to prevent falls but identified they lacked knowledge about why falls occurred and appreciated the clear and simple information presented in the programme. The education resources (message cards and video) were deemed sensible, important and very good motivational reminders but needed aesthetic and cultural revisions. The revised resources scored above > 87.3% for understandability and actionability, being synonymous with high‐quality.

**Conclusion:**

Consumers and stakeholders greatly valued the co‐design experience and contributed to the successful revision of a falls prevention education programme. The revised programme will be evaluated in a larger research trial aimed at preventing falls and enabling older homecare clients to remain safely at home.

**Patient or Public Contribution:**

The older consumers who participated in this study were recipients of homecare support services with lived experience of falls. They contributed to the revision and co‐production of a novel falls prevention education programme that originated through consumer and community involvement. The research team includes a consumer investigator and co‐author (BJ) who provided feedback and input at all stages of the research, including project design, data analysis and manuscript review.

**Abbreviations**
CALD: culturally and linguistically diverse
CCI: consumer and community involvement
CI: confidence intervals
PEMAT‐AV: Patient Education Materials Assessment Tool for audiovisual materials
PEMAT‐P: Patient Education Materials Assessment Tool for printable materials

## 1 Introduction

Falls remain one of the most pervasive and preventable threats to the health and independence of older adults. Older community‐dwelling clients receiving homecare services experience a 50% higher rate of falls than age‐matched peers not receiving services [1]. The repercussions of falls can extend beyond immediate injury, leading to functional decline, hospitalisation and premature admission to permanent residential aged care [2]. Despite this burden, preventive responses remain inadequate; only 27% of older homecare clients who sustained a fall were referred to falls prevention programmes [1]. This gap between risk and intervention underscores an urgent need to improve opportunities for homecare clients to participate in falls prevention.

In Australia, community homecare refers to government‐subsidised services designed to support older people with more complex care needs to live independently and maintain their wellbeing at home. These services may include nursing, therapy, domestic assistance, and social support [3]. However, falls prevention programmes are not routinely embedded within standard homecare services. World falls guidelines recommend all community dwelling older people receive education to raise awareness and understanding of their personal falls risk factors and how to mitigate them [4]. Incorporating falls prevention education within existing homecare services is therefore both evidence‐based and aligned with Australian Government priorities to strengthen outcomes for older people receiving care at home [5].

Embedding consumer and community involvement (CCI) in research is vitally important in shaping decisions that impact relevance and outcomes [6]. Our team have worked closely with consumers and stakeholders in hospital, residential aged care and community settings in co‐designing falls prevention education programmes [7, 8, 9, 10, 11]. Capturing consumer lived experiences and perspectives directly informs programme development, content and implementation to optimise success. Our novel falls prevention education programme, developed with older people for delivery by staff went through two prior revisions and evaluations. First, co‐designing and evaluating the programme with older people in residential aged care supported positive behaviour change and highlighted the need for autonomy in both programme resource format and aesthetics to promote engagement [8]. Second, co‐design and evaluation with older people living with dementia and their caregivers resulted in clarification of older people's awareness of how to prevent falls, and alleviated caregiver concerns regarding the person they cared for falling [11].

The programme comprised of safety messages addressing a range of falls risk factors paired with a strategic action framed in rhyme as a memory prompt [8, 11]. For example, age‐related changes to the eyes can make vision challenging, particularly in low light conditions, making moving around safely a fall risk. The safety message to mitigate this risk is, ‘*If it's not nice and bright, I'll put on a light*’ supported with brief explanatory text and photograph. Two key resources, based on client preferences were co‐produced: an 8‐min video and printable cards, both containing the safety messages with actions to mitigate the falls risks. These resources addressed visual, auditory, reading, kinaesthetic and multimodal learning style preferences [12].

However, successful implementation requires tailoring an intervention to the local context. For homecare clients, this means ensuring that programme content, resources and delivery align with the needs of both the older people receiving services and the staff providing them [1, 11]. Therefore, the aim of this study was to revise a novel falls prevention education programme with older homecare clients (consumers) and staff (stakeholders) to facilitate implementation in the homecare setting.

## 2 Materials & Methods

### 2.1 Design

A mixed methods design underpinned by a community‐based participatory research approach [13], with focus groups to provide a range of perspectives for guiding revision of the education programme to meet consumer and stakeholder needs [14]. We followed a six‐step co‐design framework with consumers and stakeholders comprising the following processes: 1. ‘*engage and align*’ to plan falls prevention education together 2. ‘*explore and connect*’ to understand experiential thoughts on falls and falls prevention 3. ‘*imagine and decide*’ to generate ideas for the falls prevention education programme, including content and design 4. ‘*create and test*’ to revise the falls prevention education resources and ascertain suitability for implementation 5. ‘*co‐implement and co‐evaluate*’ to conduct the programme and determine the impact of the falls prevention education 6. ‘*share and celebrate*’ to disseminate findings with the broader community [15]. This study reports on steps 1‐4 and is part of a larger implementation‐effectiveness trial aimed at enabling frail, older adults to prevent falls and remain safe and mobile at home [16] Figure 1.

**Figure 1:**
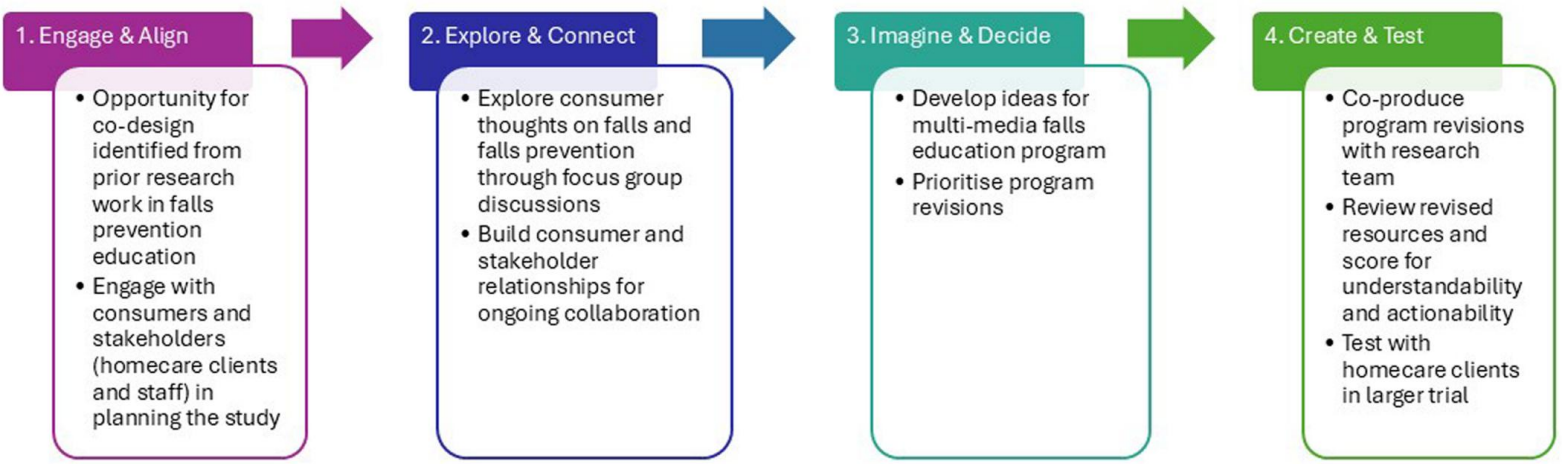
Overview of co‐design framework steps 1–4.

### 2.2 Ethical Considerations

Ethical approval for the study was received from the University of Western Australia Human Research Ethics Committee (2023/ET000763) and the participating organisation's executive directorate. All participants provided written informed consent.

### 2.3 Participants and Setting

This study partnered university researchers with consumers (homecare clients) and stakeholders (homecare staff) from a large not‐for‐profit homecare organisation in metropolitan Perth, Western Australia. The organisation provides services such as home support (e.g. personal care and cleaning), clinical support (Nursing, Allied Health) and social support (companionship, shopping, transport) to 575 clients. Consumers were eligible if they were aged ≥ 65 years, receiving homecare support services at the time of consent, able to read and communicate in English and provide written informed consent. Stakeholders were eligible if they were aged ≥ 18 years, employed in homecare client management or service delivery by the organisation for a minimum of 1 month.

### 2.4 Recruitment

A purposive sampling strategy was used to capture consumer and stakeholder characteristics including age, gender, ethnicity and staff role. The participating organisation identified potential consumers and stakeholder staff from its electronic records and referred them to the research team. The researchers screened potential participants to address the sample variation requirements. Eligible participants (consumers and stakeholders) received verbal and written information by telephone and email, had the opportunity to clarify queries and provided verbal consent prior to enrolment.

### 2.5 Data Collection and Procedure

Focus groups were conducted in a private meeting room at the organisation's central administration building, lasting approximately 1–1.5 h with a maximum of six participants in each, and followed the method described by Liamputtong [14]. Participants were greeted by the researchers and signed the consent form. The focus groups followed a guide with questions and prompts to ascertain participants awareness, thoughts and opinions about falls and falls prevention (consumers only) and the education resources (see supplementary file 1a). All groups were audio recorded. Two members of the research team (VJ, JFC) shared responsibility for moderation, observation and note taking. Notes were documented on a password protected laptop computer by a member of the research team. Discussion topics included knowledge and awareness of falls and falls prevention, views on falls education, resource content and design preferences. Participants were shown the falls prevention education video on a large screen TV and paper copies of the 16 A4 safety message cards (see Supplementary File 1b).

Consumer and stakeholder feedback guided the research team in making revisions to the programme resources. Participants were then shown the revised resources [printable safety message cards and video] and completed the Patient Education Materials Assessment Tool for printable materials and audiovisual materials respectively (PEMAT‐P and PEMAT‐AV) before providing any final verbal comments [17]. Stakeholders were advised to consider PEMAT items from a client perspective.

The PEMAT assesses the understandability and actionability of a resource and includes items such as purpose, language, content organisation, design, clarity, imagery and actions. PEMAT scores of ≤ 70% suggest the printed or audio‐visual resources are considered poorly understandable or poorly actionable [17]. At the close of each focus group member checking was undertaken to enhance confirmability and credibility [14]. All consumers received an honorarium in recognition of their time, and consumer contribution was acknowledged with a certificate of participation and co‐authorship of this manuscript.

### 2.6 Data Analysis

Demographic and survey data were summarised using descriptive statistics in SPSS version 29 statistical software (IBM SPSS Inc., Chicago, IL, USA). Quantitative data from PEMAT‐P and PEMAT‐AV were normally distributed and analysed using one‐sample t‐tests, with findings presented as means and 95% confidence intervals (CI). Qualitative data from audio recordings were transcribed verbatim, combined with researcher notes and managed in NVivo (QSR International Pty Ltd.). All transcripts were read multiple times by the researchers for familiarity and accuracy. Deductive content analysis was selected as the analysis was structured around pre‐defined categories that were already known [18]. Consumer thoughts on falls and falls prevention, together with consumer and stakeholder thoughts on falls education and the multi‐media resources were mapped to the co‐design process framework by constructing a category matrix using the criteria describing ‘*engage and align*’, ‘*explore and connect*’, ‘*imagine and decide’* and ‘*create and test*’ [15]. This study was conducted in accordance with the GRIPP‐2 Short Form checklist for reporting patient and public involvement in research (see Supplementary File 1c) [19].

## 3 Results

Findings are described using the consumer evaluation framework, consumer voices are denoted by the letter ‘C’ and stakeholders by the letter ‘S’.

### 3.1 Engage and Align

Twelve homecare clients and eleven staff (*n* = 23) participated in revising education to prevent falls at home across four focus groups. The mean age of consumers was 79 years and staff 42 years, with 82.6% (*n* = 19) being female (see Table 1).

**Table 1:** Participant characteristics.

Characteristics	Consumers (*n* = 12)	Stakeholders (*n* = 11)
Age (mean, SD) years	79 (± 6)	42 (± 12)
Age (median, range) years	81 (75‐84)	41 (31‐50)
Female sex, n (%)	10 (83.3)	9 (81.8)
Ethnicity, n (%)	0 (0)	3 (27.3)
Staff Role, n (%):		
Allied health professional		3 (27.3)
Management staff		3 (27.3)
Community support worker		3 (27.3)
Community therapy assistant		2 (18.2)

#### 3.1.1 Consumers

Consumers were keen to let the research team know how pleased they were to be part of a research project that was meaningful to them, that they were listened to, and their opinions were valued, C11 responded ‘*I thought it was great that the university was reaching out to older people like us*’ and C5 confirmed *‘I particularly appreciated knowing that whatever contribution I could make was valued…again, not always common experience. And a bit of a thrill when the resources came out and I saw some of my suggestions implemented!’*

#### 3.1.2 Staff

Staff were equally pleased to make a contribution to a topic of importance to their clients, S11 added *‘It's really important for them to know about* [falls prevention] *and to take some action’*.

### 3.2 Explore and Connect

#### 3.2.1 Consumers Only

When consumers were asked about their thoughts on falls they described their feelings and the traumatic impact of the experience, C2 reflected, *‘They* (falls) *hurt! I had two in the last year…you feel stupid that you've done it*’ C9 added, ‘*You usually damage yourself with the fall…maybe it's the beginning of the end’* and C8 concluded, *‘I think you take a longer time to heal now. It's more serious than you might imagi*ne’. Many consumers expressed uncertainty regarding what caused a fall, C10 recollected, ‘*I fell just for no reason…walking along the road*’ and C5 noted, ‘*an important part of falls for me, is knowing why they happen…and we often don't know…we want to*’. Consumers were most comfortable ascribing falls to environmental hazards C10, ‘*There's an awful lot of uneven surfaces around this village*’ and C9 ‘*Winding yourself up with the lead of the vacuum cleaner!*’ Only a few consumers indicated awareness of intrinsic falls risk factors linked to impaired strength and balance C2 stated, ‘*I skipped through the door and didn't lift me foot high enough!*’ and C8, ‘*Some people…putting their underwear on, they just can't stand on one leg, but they think they still can’*. Consumers showed awareness of some of the strategies for preventing falls C2 reflected, ‘*I have to stop being on automatic…I need to pause, to plan and then move off these days’* C1 declared, ‘*watch where you are walking*’ and C6 added, ‘*don't try and carry too much*’. Taking a proactive approach to prevention was perceived as beneficial by some consumers, C8 stated, ‘*Exercising…that's the key to not falling ‐ good balance*’, C4 described advice they would give, ‘*she has a rug…if she was a relative of mine, I would say* (name) *get rid of that!’* and C2 added, ‘*Get your cataracts done!…I couldn't see clearly’*. Consumers also voiced their concerns regarding falling with C2 summing up, *‘Don't want another fall because maybe next time the bone will break and that's it; then I'm wheelchair bound for the rest of my life. So, it's prevention, prevention, prevention!’*

### 3.3 Imagine and Decide

#### 3.3.1 Consumers

All consumers strongly related to the safety messages on card and video and elaborated on their experiences of navigating falls risk factors through their lived experience. For example, one consumer shared a confirmation about the importance of having enough light to walk safely, particularly at night (C1), *‘This is interesting about the lights, nice and bright and putting on the light. The place that we were staying each of the rooms had a little sensor light…that plugged into the socket just this soft little glow came on, there was enough vision for you to sort of move around, so we're actually going to go out to buy some*.’ The utility of messaging as prompts to take action was universally reiterated, C5 *‘They're very, very good reminders…very sensible…jog the memory, when the memory forgets’* and C6 *‘they are all very common‐sense ideas…we need reminding’ and C12, ‘It's not telling us to do things that are not possible.’* However, there was agreement amongst participants that having knowledge about risk factors didn't necessarily result in action, C6 summed up *‘There was nothing there that I really didn't know…but I don't practice’*. Perceived barriers to engaging with the programme resources related to identity, C2 commented *‘People don't want to admit that they're at that level…of needing help’* whilst others felt attitude was a contributor C4 *‘I think it's attitude…with interfering with my life’ and* C5 *‘some people just aren't open to education, not just the know‐it‐alls’*. Conversely, being open and inquisitive was perceived as an enabler C5 added, *‘Some of us have a more open and inquiring mind and that makes it easier to take in new information’*.

Consumers provided detailed feedback on resource design. There were strong opinions on message card aesthetics with contrast and colour changes suggested C9, ‘*The dark compared to the background…because often you just can't read something if there's not enough contrast*’ and including a blue/green colour was favoured C2 explained, ‘*it's calming…not distressing*.’ The amount of text was deemed important as not to overwhelm C5 commented, ‘*Some have quite a lot of words…three points are good, six points are too much’* and C3, ‘*Too many, you just turn off*’ and the value of text supported by photographs ‘*it's nice to have the picture…imagine if you just had all these words*’. Consumers were not keen on the landscape layout of the message cards with the photograph and text side‐by‐side C10 explained, ‘*When you look at the rhyme, then photo…you may not read the side message*.’ There were mostly compliments regarding the video C1 reflected, ‘*I found that very engaging even though the material was exactly what we've read [on the cards] it reinforces the written word*,’ C4 added ‘*it captured my attention and kept it*’ and C6 affirmed, ‘*Good length, I didn't get bored watching it*.’ Concern was raised about one clip of a client walking downstairs without using a handrail C3 commented, ‘*There's not much mention of handrails*’ and C4 supported, ‘*I think rails are important* (for safety)*…if you want a bit added*.’

#### 3.3.2 Stakeholders

Stakeholders valued the content appropriateness and educational principles used in the resources S8 affirmed, ‘*I do like the fact that it's, you know, common risk factors that a lot of people that we deal with would have*’ and S6 stressed, ‘*it's really important for them to know about* (falls risk factors) *and to take some action*.’ From a client learning perspective S4 shared, ‘*I like the rhymes*…*that's the kind of catch them…something that actually makes them look at it and go, ah yeah, and then read*’ and S2 pointed out, ‘…*having instructions, step‐by‐step*.’ Image diversity was a strong point for improvement amongst stakeholders S4 stated, ‘*One thing I will mention though is possibly the lack of diversity, in representation of people of different cultures and backgrounds*’ and S7 added, ‘*because that image of the very obviously white middle class couple, is not a reflection of a portion of our clients.’*

The image quality of the message cards was deemed ‘*a bit grainy*’ and text spacing slightly overcrowded S3 stated, ‘*I would like to see a little bit more of gap in between*’. One stakeholder (S9) drew on his personal experience for preparing client materials recommending a website for making design improvements, ‘*There's a website called CANVA…it would really add, help with the aesthetics side of things*.’ Most stakeholders felt the video was good quality viewing S5 reflected, *‘I felt like the way that it was filmed, the way that you whited out, the level of the volume and the titles that you laid over it, allowed you to really focus on the point. It was really well done’* and S7 added, ‘*I think it's really clear. You know, it's done with, I think a little bit of humour… I think it's lovely to watch.’* One stakeholder also pointed out that some clients’ may prefer to just watch the safety messages that addressed their personal falls risk factors so having an indication of where each message was sequenced could be helpful.

### 3.4 Create and Test

Table 2 summarises programme revisions based on consumers and stakeholders recommendations. Imagery, content, message card design and format were all perceived improvements as voiced by consumers and stakeholders.

**Table 2:** Revisions to programme resources.

Resource	Revision type	Supporting quotations
Safety message cards	Re‐shot image of CaLD clients to be included in 6 safety messages for improved inclusivity	*‘Now you can offer them with different cultural image*s…*that would probably help*.’ S7
	Re‐designed cards in A4 portrait format for improved readability	*‘The format, oh that's good’ C12 ‘then you read the message that's below it’* C10
	Re‐designed cards with white background and contrast black text with blue border trim for improved readability	*‘So that does stand out a lot more [agreement] that's better for people who might have difficulties seeing’* C10
	Increased resolution of images for improved visual engagement	*‘Clearer! It's not a blur… grabs my attention’* C5
	Reduced the amount of text and points in messages to minimise information overload	*‘Yes, concise 1‐2‐3 points!’* C3
Video	Re‐shot 4 safety messages to include client from CaLD community messages for improved inclusivity	*‘I think the images are really thorough now’* S7
	Re‐shot safety message of sufficient lighting to include client holding handrail descending stairs for improved safety	*‘10 out of 10! very helpful…hanging on to a rail to go downstairs’* C3
	Timestamps were added to the video offering the option to skip to clients’ personalised safety message(s)	*‘If I were to use the video I would only show the parts that are relevant’* S9

The revised resources co‐created by consumers, stakeholders and researchers achieved total PEMAT suitability scores ≥ 87.3%, with a top score of 96.7% (Table 3), which is synonymous with a high‐quality patient resource. The message cards received the highest understandability score (96.1%) from consumers and the lowest actionability score (76.4%) from stakeholders. The video was rated highly on usability by consumers (93.9%) and stakeholders (95.8%) and achieved 100% rating for actionability.

**Table 3:** PEMAT‐P and PEMAT‐AV scores for revised programme resources.

	Consumers (*n* = 12)	Stakeholders (*n* = 11)
PEMAT‐P^a^ (Revised safety message cards)		
Total score, *M* (*95%CI*)	19 (17.9, 19.8)	17.5 (15.1, 19.8)
Total percentage, *M* (*95%CI*)	94.2 (89.5, 98.8)	87.3 (75.5, 99.0)
Subscale of PEMAT‐P		
*(i)Understandability*		
Total score, *M* (*95%CI*)	14.4 (13.8, 15.0)	13.6 (12.1,15.1)
Total percentage, *M* (*95%CI*)	96.1 (92.3, 99.9)	90.9 (80.8, 100.0)
*(ii)Actionability*		
Total score, *M* (*95%CI*)	4.4 (3.9, 4.9)	3.8 (2.7, 4.9)^b^
Total percentage, *M* (*95%CI*)	88.3 (78.3, 98.4)	76.4 (54.9, 97.9)^b^
PEMAT‐AV^c^ (Revised safety message video)		
Total score, *M* (*95%CI*)	13.3 (12.8, 13.8)	13.5 (13.2, 13.9)
Total percentage, *M* (*95%CI*)	95.3 (91.7, 98.8)	96.7 (94.3, 99.3)
Subscale of PEMAT‐AV		
*(i)Understandability*		
Total score, *M* (*95%CI*)	10.3 (9.8, 10.3)	10.5 (10.2, 10.9)
Total percentage, *M* (*95%CI*)	93.9 (89.4, 98.4)	95.8 (92.7, 99.1)
*(ii)Actionability*		
Total score, *M* (*95%CI*)	3^d^	3^d^
Total percentage, *M* (*95%CI*)	100^d^	100^d^

*Note: M* Mean, *CI:* Confidence Interval

^a^ Only 20 questions were relevant to our printed materials (15 questions pertaining to Understandability and five pertaining to Actionability).

^b^ One participant did not answer all actionability questions.

^c^ Only 14 questions were relevant to the video (11 questions related to understandability and three questions for actionability).

^d^ No 95%CI (no standard deviation) as all participants agreed with all three questions in both groups.

## 4 Discussion

This study engaged consumers and stakeholders in the co‐design of a falls prevention education programme for older homecare clients, addressing a critical gap as little co‐design research has previously been conducted in homecare settings. Findings provide a compelling case for integrating the voice of the consumer into the design and delivery of falls prevention programmes as articulated by the principle of ‘*nothing about us, without us*.’ This advocates for the meaningful engagement of older people in shaping interventions that affect their daily lives as being essential not optional [20]. By embedding a consumer co‐design framework, we demonstrated how consumer voices directly shaped programme refinement, consistent with recommendations for meaningful CCI in preventive health [21].

Co‐design can ensure that educational materials are not only evidence‐based but also relevant and meaningful to the people who will use them. This is particularly important when developing falls prevention education for older adults receiving care at home, as individual differences in functional capacity, health literacy, preferences and living circumstances may influence how information is understood and acted upon. Engaging older people in the development process provides an opportunity to identify preferred content, language, imagery and formats and to adapt education to their everyday experiences. Evidence indicates that older adults value clearly structured information, larger text, relevant visual material and accessible formats, while falls‐prevention studies demonstrate that co‐designed resources can be perceived as enjoyable and informative and can incorporate preferences that may facilitate engagement [8, 22].

A central finding was that whilst many consumers understood the devastating impact of falls, they were uncertain about the causes, highlighting a persistent knowledge gap that educational programmes need to address. Understanding what older people think about falls and falls prevention is a vital step for designing falls prevention programmes older people are likely to engage with [23]. The importance of raising awareness and understanding of personal falls risk factors and how to mitigate them are key recommendations in global falls prevention guidelines [4]. Our programme safety messages provide education on a range of intrinsic and extrinsic falls risk factors, explaining why falls happen and what actions can be taken to prevent them [8, 11]. This is important for research translation, as a scoping review on older adult's knowledge of falls risks and prevention found that improving older people's understanding of their risk of falling, and emphasising the fact that most falls are preventable, may help motivate older people to take preventative action [23].

The role of environmental hazards was strongly endorsed by consumers, who identified lighting, clutter, and lack of handrail use as easily modifiable falls risks. These findings align with Cochrane evidence indicating that approximately 30% of falls in the home are attributable to environmental hazards [24]. Consumers valued the environmental safety messages in our education resources that outlined how to improve lighting and walkways. Most importantly they co‐designed the use of handrails when negotiating stairs for improved safety. Given that our consumers represent a frailer community cohort ‘at higher risk’ of having a fall compared with aged‐matched peers [1], these insights are of salience. Similarly, a Cochrane review on environmental interventions for preventing falls in high‐risk community dwelling older people found addressing environmental hazards such as poor lighting, clutter and stairs without rails can reduce the number of falls by 38% [24].

Consumer and stakeholder feedback showed high levels of agreement that the video was easily understood and would assist homecare clients to take actions to reduce their risk of falling. This aligns with findings of a review of video‐based health education in which videos were shown to have superior effectiveness over other educational methods in patient uptake and health literacy, when conferred in an understandable way [25]. In terms of implementation, this finding suggests it could be important for staff to emphasise to homecare clients the importance of watching the safety message video as it may be more likely to encourage them to take the necessary actions to reduce their risk of falling.

### 4.1 Strengths and Limitations

A key strength of this study was the active involvement of both consumers and stakeholders, which fostered authentic co‐design and ensured resources reflected the lived experiences of those most at risk of falling. The use of small focus groups promoted interactive discussion and facilitated deeper insights into awareness, barriers, and preferred education design.

However, findings from our study are limited to a single community homecare organisation in Western Australia and despite active recruitment, participants representing culturally and linguistically diverse (CALD) groups was low. We plan to have a more focused CCI partnership with CALD groups in future work, as authors are aware the design and delivery of effective health education requires the assimilation of cultural preferences as well as language translation [26]. This study explicitly focused on steps 1–4 of the co‐design framework. Step 5 (*Co‐implement and Co‐evaluate*) is currently being addressed in an implementation‐effectiveness trial [16], and step 6 (*Share and Celebrate*) will involve dissemination of findings with consumers, stakeholders, and the broader community through local community meetings, manuscript publications and presentations.

## 5 Conclusion

Consumers and stakeholders greatly valued the opportunity of contributing to the co‐design of a falls prevention education programme for homecare clients, drawing on their lived experiences, that resulted in the production of high‐quality, tailored resources for evaluation in a larger research trial. Consumer‐led co‐design can bridge the gap between evidence about falls prevention and its successful implementation into the intended population, ensuring falls prevention education is relevant, inclusive, and effective for those most at risk.

## Author Contributions

**Jacqueline Francis‐Coad:** conceptualisation, funding acquisition, writing – original draft, writing – review and editing, formal analysis, resources, methodology, investigation. **Jo‐Aine Hang:** writing – review and editing, formal analysis. **Vanessa Jessup:** project administration, investigation, writing – review and editing, data curation, formal analysis. **Sharmila Vaz:** writing – review and editing. **Elissa Burton:** writing – review and editing. **Cheng Yen Loo:** writing – review and editing. **Bethwyn Joy:** investigation, writing – review and editing, data curation, formal analysis, conceptualisation. **Anne‐Marie Hill:** conceptualisation;, writing – review and editing; funding acquisition.

## Conflicts of Interest

The authors declare no conflicts of interest.

## Supporting information


Supplementary File

## Data Availability

All data generated or analysed during this study are included in this published article [and its supporting information files].
