# Supplementary material for: Working Together With Consumers to Develop Falls Prevention Education for Older People Receiving Homecare – a Community‐Based Participatory Study
Source: Health Expect. 2026 Oct 26;29(5):e70948. doi: 10.1111/hex.70948 (PMC13700112; doi:10.1111/hex.70948)
Supplement: Supplementary file 1 [file HEX-29-e70948-s001.docx]

**Supplementary file 1**

**a. Focus group guide**

*Welcome & introduction:*

We have the discussion scheduled for approximately 1-1.5 hours today. During the group, we want to obtain your views as consumers/stakeholders with regard to a falls prevention program that can be delivered at home’.

My role is to facilitate the session today. Please feel free to discuss or raise any issues of importance to you personally and / or to your colleagues. There are no right or wrong answers here. Regardless of any opinions you give, your statements will not be shared in an identifiable form outside the investigator group. The approved investigators, who will help to review your feedback, only so as to improve our program and service for future home care clients. We are interested in hearing your point of view even if it disagrees with others’ opinions.

It is my role to keep the discussion focused on the topic we are here to discuss, so I may need to move the conversation along so we can cover all the items and to make sure that we get to hear from everyone here today.

It is important that we allow others to provide their feedback without challenge and respect others’ beliefs and opinions.

We will be audiotaping this discussion, with your permission, because we don’t want to miss any comments. It is important for you to realise that no names will be attached to the reports arising from this work or any publications. You may be assured of complete confidentiality in the report and publications.

Before we start the group I would like to emphasise the need for respect and confidentiality and the importance of hearing everyone’s views. I would ask respectfully that one person speaks at a time and to speak up for the audio-tape.

I would like to introduce you to my colleagues (moderator/notetaker)

Ice breaker ☐

I would like you to introduce yourselves…

*Focus Group Data Collection Template*

Key: Yellow highlight consumers only

| **Focus Question Guide** | **Responses** | **Key Issues** | **Review note** |
| --- | --- | --- | --- |
| **Intro Qu (brief)** | | | |
| What do you know about falls? Prompt: Causes |  |  |  |
| What do you know about falls prevention? |  |  |  |
| **Transition Qu** |  |  |  |
| Please tell me more… |  |  |  |
| **Focus Qu** |  |  |  |
| Introduce education resource materials | | | |
| What are your thoughts on the fall prevention safety messages? (and card format)  Was there anything new for you?  What did you find  interesting? Why? |  |  |  |
| What are your thoughts on displaying the message cards as reminders around your home? If no why? What would you prefer? |  |  |  |
| What are your thoughts on the video? How would you prefer to watch the video? Prompt: Have a link to online view? Download a copy onto a device? |  |  |  |
| Would you recommend these resources to others? |  |  |  |
| **Short summary by moderator** | | | |
| Check interpretation of discussion | | | |
| **Concluding Qu** |  |  |  |
| Is there anything else you wish to add or comment? |  |  |  |
| **Final Reflections** |  |  |  |

Thank you all for coming today we really appreciate your feedback.

b. Safety messages & sample message card

|  | **Rhyming message and Explanation** |
| --- | --- |
| 1 | **“Feeling unwell or sick? Alert someone quick”**   - If you feel unwell, dizzy or a bit unsteady tell your partner or support worker immediately - Getting checked early may prevent an illness developing or a fall from happening - If you have a personal pendant alarm use it to gain timely assistance |
| 2 | **“My walking aid is near and my pathway is clear”**   - Check your walking aid is within reach at all times - Have a look around in the direction you are planning to walk to check your pathway is clear - Ask your Physiotherapist to show you the best way to walk using your walking aid - Add a bell to the collar of your small pets so you know if they’re close by |
| 3 | **“There’s nothing to fear if our pathway is clear”**   - Have a look around in the direction you are planning to walk to check your pathway is clear - Add a bell to the collar of your small pets so you know if they’re close by - If you have any concerns about your walking talk to your Physiotherapist |
| 4 | **“When hazards are removed our safety is improved”**   - Check around your home for hazards like loose mats, curled carpet edges or wet bathroom floors that may cause you to slip or trip - Check outdoor verandas or paths for plant debris or uneven surfaces - Ask for assistance to remove or replace any potentially dangerous items |
| 5 | **“We need good sight to see clearly day and night”**   - Age-changes and eye disease may blur or limit our field of vision - Make sure you always wear your prescribed glasses when you are moving around - Clean your glasses regularly - Have your eye-sight checked regularly with your Optometrist or Doctor |
| 6 | **“If it’s not nice and bright we’ll put on a light”**   - Changes to our eyes as we age mean that approximately 3 times the amount of light is required to see the same as a younger adult - This makes it harder to see your environment as clearly when light levels are low - So switch on the light |
| 7 | **“We’ll switch on a light if we get up at night”**   - Many of us need to get up during the night, most commonly to visit the toilet - Lighting is important to help us walk safely at night - Lighting solutions that won’t disturb our partner include bedside touch lamps or sensor lighting strips - Allow time for your eyes to adjust to your lighting level |
| 8 | **“We’ll choose with care the clothes we wear”**   - Choosing well-fitting fashionable clothing will allow you to move freely - Checking and adjusting clothing so that hemlines aren’t dragging on the ground will help avoid tripping or slipping |
| 9 | **“Our supportive shoes are the ones we’ll use”**   - Choose shoes (and slippers) that fit well, with good adjustable support to accommodate swelling - Soles that are thinner will help you navigate the ground beneath your feet - Avoid walking around in stockings or socks only - Have regular foot checks, talk to your Podiatrist or Doctor |
| 10 | **“When changing position staying steady is our mission”**   - Pause when changing from one position to another, like standing up from a chair or getting up from bed - This allows our blood pressure time to adjust - If you feel unsteady or light-headed sit back down and call your partner or support worker for assistance - If you are experiencing frequent dizziness, talk with your Doctor |
| 11 | **“Being steadier and stronger keeps us mobile for longer”**   - Having better balance and stronger muscles helps us to stay steady and move around well - Ask your Physiotherapist about exercises that might help you to feel steadier and stronger - Simple things like repeated sitting to standing from a chair and being a little less sedentary during the day can all help |
| 12 | **“Having enough Vitamin D is helpful for me”**   - We need vitamin D to help our muscles and bones stay strong - Our levels of Vitamin D may be lower as we get older as our skin becomes less efficient at making vitamin D from sun exposure - Taking a vitamin D supplement can be helpful if your levels are low, so check with your Doctor |
| 13 | **“Eat a healthy meal with nutritional appeal”**   - Eating a variety of foods will help fuel our bodies and promote repair - Try not to skip meals - prepare extra portions of food that can be frozen and used when you don’t feel like cooking - If you have any concerns about your eating habits talk to your Doctor or Dietician |
| 14 | **“Knowing our medications can avoid complications”**   - The number and type of medications you take can have side effects that may make you feel drowsy or unsteady on your feet - Tell your partner or support worker if you notice any changes when taking your medications - Seek advice from your Pharmacist or Doctor |
| 15 | **“Don’t get thirsty before you think it’s time to have a drink”**   - Our ability to detect when we need to drink decreases as we age - Have regular drinks throughout the day to keep hydrated - Water is best (not just tea or coffee at meal times) - Keep a water bottle or jug of water and glass close by |
| 16 | **“Getting safely to the loo is something we plan to do”**   - Try to plan enough time to walk to the toilet at your safe pace - If you have any concerns about your bladder or bowels **help is available** - Talk with your Doctor, Physiotherapist or Support worker |

c. GRIPP2 Checklist

| Section and topic | Item | Reported on page No |
| --- | --- | --- |
| 1: Aim | Report the aim of PPI in the study | Consumer involvement stated in Aim pp.4,5 |
| 2: Methods | Provide a clear description of the methods used for PPI in the study | Design used community-based participatory approach and a co-design framework pp. 6-8, figure 1 |
| 3: Study results | Outcomes—Report the results of PPI in the study, including both positive and negative outcomes | Results reported using co-design framework pp. 9-14 |
| 4: Discussion and conclusions | Outcomes—Comment on the extent to which PPI influenced the study overall. Describe positive and negative effects | Discussion/Conclusion report positive consumer impact and facilitators for implementation pp. 14-17 |
| 5: Reflections/critical perspective | Comment critically on the study, reflecting on the things that went well and those that did not, so others can learn from this experience | Strengths and Limitations section comment critically on the study with reflections and learnings pp. 16,17 |
